# Multimodal imaging assessment for feasibility of endovascular reconstruction in cases of inferior vena cava atresia

**DOI:** 10.1002/ccr3.8831

**Published:** 2024-05-24

**Authors:** Adam Kisling, Sean Basile, Adelle Dagher, Jonathan Sexton, Eric Twerdahl

**Affiliations:** ^1^ Department of Cardiology Walter Reed National Military Medical Center Bethesda Maryland USA; ^2^ Department of Surgery Walter Reed National Military Medical Center Bethesda Maryland USA; ^3^ Department of Vascular Surgery Walter Reed National Military Medical Center Bethesda Maryland USA

**Keywords:** CT angiography, endovascular venovenous reconstruction, inferior vena cava agenesis, inferior vena cava atresia, invasive venography

## Abstract

Inferior vena cava atresia is a rare condition with highly variable anatomy due to the complexity of caval embryology. When endovascular venovenous reconstruction is considered for severe persistent sequelae, multimodality imaging with CT and invasive venography is used to determine the appropriateness of intervention and for procedural planning.

## 1 INTRODUCTION

The inferior vena cava (IVC) develops embryologically from the fusion of primordial veins: the intrahepatic IVC from a right vitelline vein, the suprarenal IVC from the right subcardinal vein, the renal IVC from the subcardinal‐supracardinal anastomosis, and the infrarenal IVC from the right supracardinal vein. IVC agenesis results from the aberrant development or fusion of one or more of these structures during the fourth to eighth weeks of gestation resulting in a complete lack of organ formation at the corresponding IVC section. IVC atresia, on the other hand, is due to a partial failure of proper development of any of the primordial veins or a secondary process such as IVC thrombosis that leads to a significant reduction in the IVC lumen.^1^ In these conditions, deoxygenated blood returns to the right atrium through a robust collateral venous system.^2^

IVC atresia is a rare condition with a prevalence of 0.0005%–1% of the population.^1^, ^2^ It can be asymptomatic due to collateral flow and discovered incidentally in approximately 25%–33% of cases.^1^, ^3^ However, patients may present with a myriad of symptoms such as painful engorgement of superficial collateral veins, lower extremity swelling, venous thrombosis (VT) at a young age, non‐healing venous ulcers, and rarely renal dysfunction. Premature development of lower extremity VT is the most common initial presentation of this condition.^2^, ^4^ Notably, IVC agenesis and atresia can have similar disease presentations and treatment strategies. There is no consensus on a standard management of IVC anomalies, which is largely due to the high degree of heterogeneity in presentation, severity of complications, and anatomy.

Anticoagulation is a mainstay of therapy to prevent loss of collateral blood supply due to thrombosis and to prevent VT in the lower extremities in the setting of congestive venous stasis. Additionally, an association between IVC atresia and hereditary thrombophilia has been reported.^5^ Given that management is primarily focused on the treatment of complications, many patients will require endovascular pharmacomechanical thrombectomy or catheter‐directed thrombolysis.^3^ Unfortunately, treatment of thrombosis alone is not a feasible monotherapy in patients who continue to have symptomatic occlusions or non‐healing venous ulcers.^2^ Open reconstruction for similar pathology in femoral and iliocaval occlusions is associated with low patency rates and early morbidity and angioplasty lacks efficacy and durability. With the advancement of imaging techniques and endovascular procedures, endovascular venovenous reconstruction (EVR) has become a first‐line therapy for symptomatic venous occlusions and has been utilized to treat presentations of symptomatic IVC agenesis.^6^ Despite this, there is no standard management of IVC anomalies due to the complexity and heterogeneity of the anatomy of IVC anomalies, and a limited number of cases have been published thus far given the rarity of the condition.

Here we present a case of symptomatic IVC atresia in which the definition of the patient's complex anatomy with multimodality venography allowed for the determination of candidacy for EVR.

## 2 CASE PRESENTATION

Following an initial presentation for acute lower extremity VT, a 21‐year‐old male underwent a lower extremity ultrasound which demonstrated extensive bilateral VT. A laboratory evaluation for hypercoagulable disorders was unremarkable. A CT with contrast demonstrated atresia of the IVC. Extensive superficial, retroperitoneal, azygos, and hemiazygos venous collaterals were present. Over the next decade, the patient developed severe lower extremity venous insufficiency and had repeated evaluations for painful abdominal varicosities, recurrent bilateral lower extremity VTs, and bilateral lower extremity venous ulcerations ultimately necessitating surgical debridement and negative pressure wound therapy in addition to catheter‐directed thrombolysis for his extensive lower extremity thromboses. These manifestations continued to escalate despite supportive care and multiple anticoagulation therapy regimens (warfarin, rivaroxaban, and apixaban). Given the persistent nature of his symptoms and failure to respond to nonsurgical options, the patient was evaluated for EVR of his IVC to relieve the underlying obstruction and subsequent venous congestion.

## 3 INVESTIGATIONS AND TREATMENT

**FIGURE 1:**
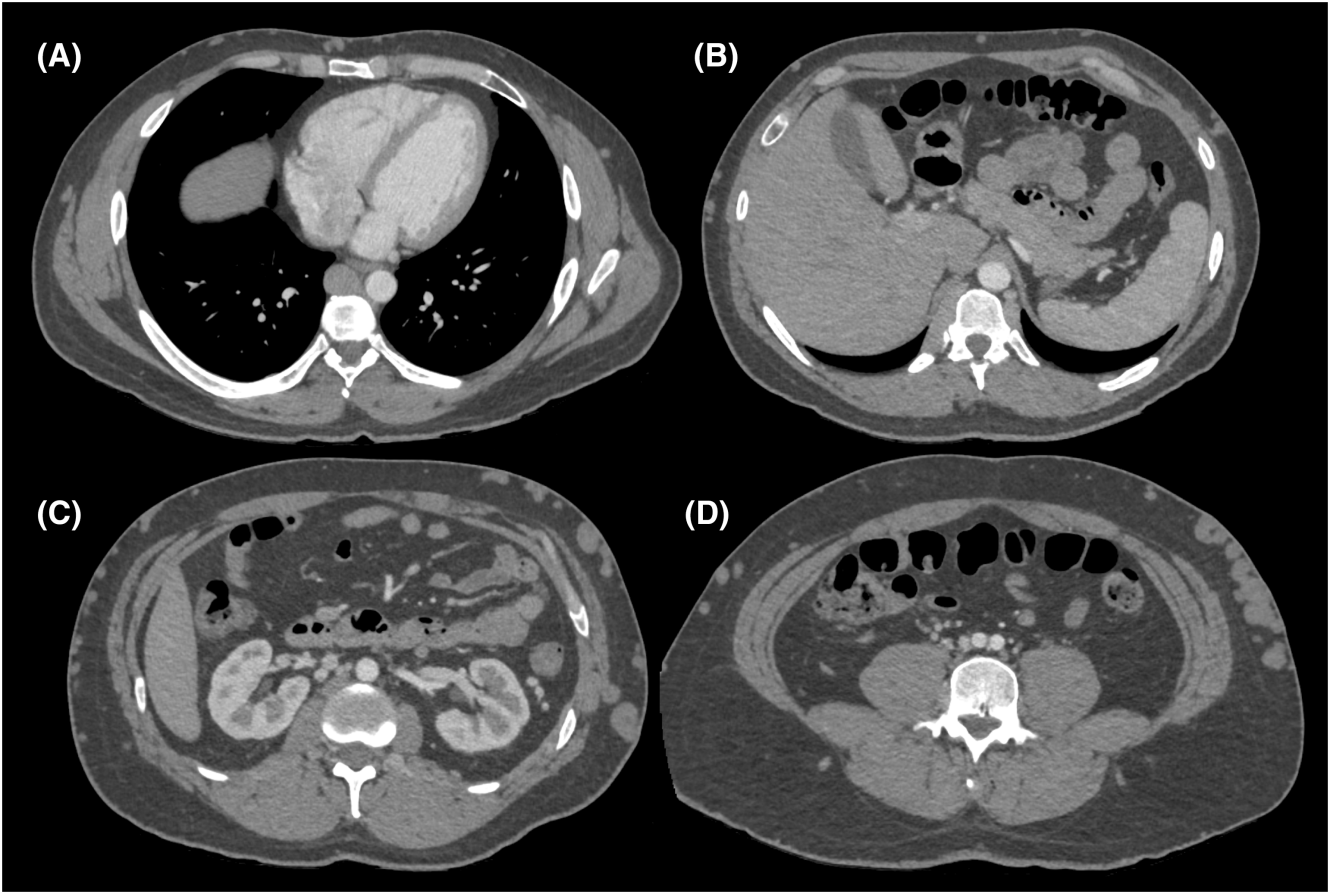
CT angiography of the chest, abdomen, and pelvis. This ECG‐gated CT was performed with a prolonged timing delay with the intention of scanning the patient when the contrast concentration was highest in the IVC. Unfortunately, due to severely delayed drainage from the lower extremities, the contrast concentration is higher in the aorta than in the IVC and its collaterals. (A) A coronal cross‐section at the level of the cardiac ventricles demonstrating an enlarged azygos vein. (B) A coronal cross‐section at the level of the liver demonstrating the region where the atretic intrahepatic IVC cannot clearly be identified. (C) A coronal cross‐section at the level of the kidneys demonstrating robust superficial collateral veins and dense intraabdominal collateral veins without a clearly identifiable true IVC. (D) A coronal cross‐section at the level of the aortic bifurcation demonstrating small iliac veins with dense surround collaterals.

The initial CT angiography (Video S1, Figure 1) showed patent iliac veins with a diminutive residual lumen of the IVC. Dense, occult renal collateral veins were noted as well as a patent superior vena cava (SVC). These findings were correlated with a recent venous duplex ultrasound. After shared decision‐making with the patient, we elected to pursue preliminary diagnostic venography to further characterize the patient's complex anatomy to allow for EVR planning and subsequent discussion of options with the patient rather than proceeding directly to EVR with venography guidance.

Initial diagnostic venography was performed through three access points: bilateral common femoral veins and the right internal jugular vein (IJV). The patient was found to have severe disease of the bilateral iliac veins with dense collateralization of the IVC extending to the diaphragm and a diminutive residual lumen of the IVC. Communication between the left iliac venous system and IVC was not demonstrated. Venography performed through the right IJV suggested the presence of a potential lumen to take for endovascular recanalization. Venography and cross‐sectional imaging identified the atretic segment of IVC which coursed from the infrarenal segment to 1–2 cm before the cavo‐atrial junction at which point the remainder of the atretic segment joining the right atrium was unable to be identified. Despite this, the decision was made to pursue EVR with the understanding that the section of poorly visualized IVC could represent an area that would necessitate blindly crossing with a guidewire which could potentially lead to severe hemorrhage or destruction of renal collateral outflows.

## 4 OUTCOME AND FOLLOW‐UP

**FIGURE 2:**
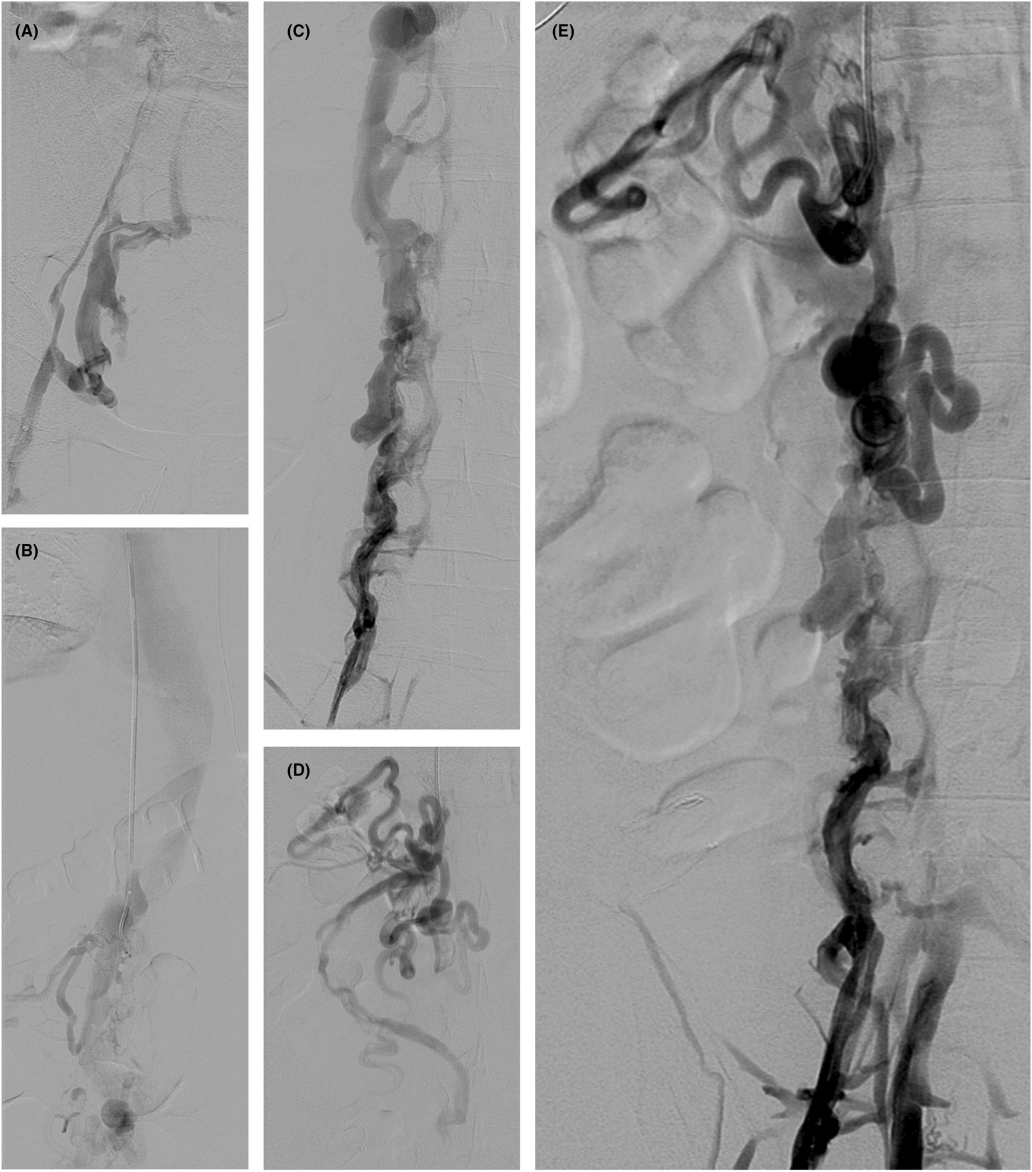
Invasive venography of IVC atresia. (A) A venogram from the left femoral vein demonstrating venous drainage from the lower extremity via superficial and retroperitoneal collaterals. (B) Late‐phase venography of the SVC and hepatic IVC. (C) A venogram of contrast draining into a dilated azygos vein which receives additional collaterals as it courses toward the SVC. (D) A venogram of the confluence of collateral vessels from abdominal organs to the hepatic segment of the IVC due to the absence of a suprarenal segment. (E) A venogram of simultaneous injection of contrast from the femoral vein and hepatic IVC.

The patient returned for the planned EVR and two access points were established at the right common femoral vein and right IJV to ensure adequate working space. Following access with micropuncture sheaths, a venogram was performed from the right common femoral vein which again highlighted the diseased right iliac system and the numerous collaterals supplying flow from the pelvis back to the IVC just below the diaphragm, all of which correlated with the findings of the prior CT angiography and venography. The micropuncture sheaths were then exchanged for five French sheaths. With careful manipulation, attempts at passing wires of varying sizes through the iliac system into the atretic IVC were made. However, no straight channel through the collaterals could be identified. The right IJV was then accessed, and a guidewire was passed down the SVC into the IVC just above the diaphragm. Venography was then performed from this area as well as from the abdominal IVC (Figure 2). This demonstrated a possible remnant vena cava medially. Despite multiple attempts to access this possible remnant, it became clear that it was not possible to pass a wire through the collaterals from the neck to the groin without blindly passing a guidewire over a long area. Due to the risks associated with blindly passing a guidewire over a long area and the inability to cannulate the torturous collateral network to reestablish inline flow, the procedure was ultimately aborted. Upon follow‐up, other invasive treatment options were considered, but the decision was made to continue supportive care only. Since that time, he has had persistence of his non‐healing venous ulcers, but he has not developed any new complications.

## 5 DISCUSSION

Management of IVC atresia is primarily medical and focuses on the treatment of complications, especially DVTs. Surgical interventions, including open venous reconstruction or EVR, may be warranted in those patients with severe symptoms refractory to other treatments.^1^, ^4^ With supportive imaging from high‐quality CT at the time of initial evaluation and invasive venography at the time of intervention as well as the advancement of endovascular reentry devices, endovascular reconstruction has become possible.^7^ Approximately 13% of patients with IVC atresia will undergo EVR.^1^ Despite the potential for significant symptom improvement and the suggested safety outcomes in published literature, careful case selection is needed due to the complexity and heterogeneity of the anatomy in cases of IVC atresia.^1^, ^6^

EVR is thought to be feasible in any case in which a wire can be safely passed across the atretic segment of the IVC. In some cases, this requires blindly passing a guidewire across a short distance between the proximal and distal ends of an obstruction seen on venography that is bypassed only by untraversable or indirect venous collaterals. In such cases, important considerations regarding feasibility include the length of the required blind crossing and the risks posed to the surrounding vascular and visceral structures by a blind cross attempt.

Many patients require judicious use of iodinated contrast due to a history of allergic‐like reactions or due to acute or chronic renal dysfunction. Particularly germane to this case is the fact that IVC atresia can lead to chronic kidney disease due to chronic renal venous congestion. In such cases, the potential harm from venography must be weighed against the symptomatic benefit that EVR may be able to provide. Premedication is an excellent option in patients with a history of allergic‐like reactions. Adequate hydration before and after contrast injection and minimization of the amount of contrast administered should be emphasized in patients with renal impairment.

Although CT angiography is a valuable initial diagnostic modality, diagnostic venography is critical in determining the feasibility and anatomic suitability of EVR.^7^ CT angiography can be technically challenging due to the difficulty of timing the study in the targeted venous phase. This difficulty is increased in cases of IVC atresia due to the unpredictability of the timing of the contrast flow through the atretic IVC and collaterals. During the evaluation for EVR, duplex ultrasound imaging is often performed to assist with defining anatomy and anticipating complications.^6^ Despite appearing accessible by collateral flow on venogram, the mechanical challenges of crossing an atretic IVC segment, even when approaching from varying venous access points, can be prohibitive of EVR. The intersection between CT angiography and comprehensive venography is paramount to identifying possible mechanical and structural contraindications in patients with severe symptoms who are refractory to all noninvasive and minimally invasive modalities and who are undergoing evaluation for EVR.

## AUTHOR CONTRIBUTIONS

**Adam Kisling:** Conceptualization; funding acquisition; project administration; supervision; visualization; writing – original draft; writing – review and editing. **Sean Basile:** Writing – original draft; writing – review and editing. **Adelle Dagher:** Writing – original draft; writing – review and editing. **Eric Twerdahl:** Conceptualization; writing – review and editing. **Jonathan Sexton:** Conceptualization; writing – review and editing.

## FUNDING INFORMATION

Partial support was received from award HU00011920029 from Defense Health Agency to the Military Cardiovascular Outcomes Research program, Uniformed Services University, Bethesda, MD.

## CONFLICT OF INTEREST STATEMENT

There is nothing relevant to disclose for any of the authors.

## CONSENT

Written informed consent was obtained from the patient to publish this report in accordance with the journal's patient consent policy.

## Supporting information


Video S1.

## Data Availability

The data that support the findings of this study are available from the corresponding author, AK, upon reasonable request.

## References

[1] Saab K , Brahmandam AS , Brackett AL , et al. Systematic review of inferior vena cava atresia. J Vasc Surg Venous Lymphat Disord. 2023;11(6):1253‐1264. doi:10.1016/j.jvsv.2023.07.002 37453547

[2] Kannappan M , Sakthi VS . Inferior vena cava agenesis: an underrated cause of deep venous thrombosis. Cureus. 2023;15:e33667. doi:10.7759/cureus.33667 36793843 PMC9924708

[3] Heafner T , Nissen AP , Schechtman D , Alderete JF , Arthurs ZM , Propper BW . Venovenous bypass using continuous renal replacement therapy and endovascular inferior vena cava reconstruction to treat bilateral massive iliocaval deep venous thrombosis. J Vasc Surg Cases Innov Tech. 2019;5(4):438‐442. doi:10.1016/j.jvscit.2019.04.004 31660468 PMC6806639

[4] Hung ML , Kwon D , Sudheendra D . Endovascular ivc reconstruction in an 18 year old patient with subtotal ivc atresia. EJVES Vascular Forum. 2021;52:5‐10. doi:10.1016/j.ejvsvf.2021.06.001 34258606 PMC8260863

[5] Rose SS , Ali Y , Bekos TJ , Saidi P , Kumar A . Deep venous thrombosis caused by inferior vena cava atresia and hereditary thrombophilia. Am J Med Sci. 2009;337(1):67‐70. doi:10.1097/MAJ.0b013e31816a8d0d 19002010

[6] Murphy EH , Johns B , Varney E , Raju S . Endovascular management of chronic total occlusions of the inferior vena cava and iliac veins. J Vasc Surg Venous Lymphat Disord. 2017;5(1):47‐59. doi:10.1016/j.jvsv.2016.07.005 27987609

[7] Scali ST , Beck AW , DeMartino RD , Duxbury A , Walsh DB . Endovascular management of congenital atresia of the infrarenal ivc. Vasc Endovasc Surg. 2010;44(3):234‐236. doi:10.1177/1538574410361790 20308176

